# Cinnamon essential oil vapor alleviates the reduction of aroma-related volatiles in cold-stored “Feicheng” peach using HS-GC-IMS

**DOI:** 10.3389/fnut.2023.1122534

**Published:** 2023-07-05

**Authors:** Dan Wang, Jing Zhang, Wen-yu Chen, Hao Zhai, Yang Jiang

**Affiliations:** ^1^Shandong Institute of Pomology, Tai’an, China; ^2^Feicheng Peach Industry Development Center, Tai’an, China; ^3^Key Laboratory of Food Processing Technology and Quality Control of Shandong Higher Education Institutes, College of Food Science and Engineering, Shandong Agricultural University, Tai’an, China

**Keywords:** aroma volatiles, flesh browning, chilling injury, cinnamon essential oil, HS-GC-IMS

## Abstract

“Feicheng” peach is popular for its unique aroma, but its defect of being highly sensitive to chilling injury (CI) often leads to aroma loss and internal browning. Essential oils (EOs) are often used to enhance the antioxidant capacity of plants and fruits, as well as to trigger their defense against biotic/abiotic stresses. This study aimed to examine the effect of cinnamon essential oil (CEO) vapor treatment on the aroma quality of peach fruit during cold storage using HS-GC-IMS. The results showed that 50 μL/L CEO vapor reduced the severity of internal browning (IB) in peaches at the stage of 7 ~ 21 d during refrigeration (Significantly, the L* value was higher and the IB index was lower than that of control, *p* < 0.05). Meanwhile, the evident reduction or loss of aroma content caused by CI was restored to a higher level than the control (*p* < 0.05). Furthermore, CEO treatment promoted the release of aroma-related volatiles as evidenced by more propyl acetate, and the dimer of amyl acetate, isoamyl acetate, butyl acetate detected than that on harvest day and no-treated group after 21 d of cold storage plus 2 d of shelf life. Genes of *PpLOX1*, *PpLOX2*, *PpHPL1* and *PpADH1* associated with aroma-related volatile biosynthesis revealed higher transcript abundance in peach fruits treated with CEO than the control (*p* < 0.05). Overall, our study demonstrated that CEO in vapor phase may be beneficial to alleviate the quality deterioration in aroma and flesh color of “Feicheng” peaches caused by CI, which lays a theoretical reference for maintaining postharvest quality of peach fruits.

## Introduction

“Feicheng” peach (*Prunuspersica* (L.) Batsch, cv. Feicheng) is a specialty fruit of Shandong Province, China. It is known as “the crown of peaches” because of its thick flesh, rich juice, rich aroma and high nutritional value. As a typical climacteric fruit, peaches ripen rapidly, releasing a wealth of volatile substances after a burst of ethylene production, creating a unique aroma. “Feicheng” peach is a kind of melting-flesh peaches, which is more likely to enter the senescence stage (softening and rotting) than stony-hard peaches at room temperature. Therefore, low-temperature storage is used to slow down the respiration of fruit and retard deteriorations. Nevertheless, prolonged cold storage can trigger a variety of physiological disorders, namely chilling injury (CI) symptoms, manifested by flesh or pit cavity browning, loss of aroma, chalking, wooly (lack of juice) and leathery (hard textured with no juice) (1–3). CI is genetically influenced and activated by a combination of storage temperature and storage period, and mainly limits the shipment and marketing of peach fruits. CI symptoms normally appear after placing fruit at room temperature following cold storage (1). For this reason, this problem is usually experienced by the consumer. Therefore, understanding and preventing the causes of CI in peaches is of economic interest.

Aroma is an important trait of “Feicheng” peach and plays an essential trait in determining the acceptability and perception of fruits products by consumers. To date, more than 100 compounds have been identified in peach and only few of them including esters, C6 aldehydes, terpenes, lactones and alcohols contribute a lot to the volatiles of peach (4–6). The volatile aroma of peach has been reported to vary by cultivars (7), processing (8), storage conditions (9), and stage maturity and/or ripening conditions (4, 10). The biosynthesis of fruit volatiles mainly derive from lipoxygenase (LOX) pathway involving various enzymes, and the expression levels of specific gene family member, *PpLOX1*, *PpHPL1* and *PpADH1*, are highly correlated with the production of volatile ester and lactone in ripening peach fruit (11). In actual production, CI leads to a decrease in the aroma of “Feicheng” peach, which greatly reduces consumer acceptance and brings serious economic consequences to the peach fruit industry (12). However, few studies have been conducted on the changes of the volatiles within the “Feicheng” peach during cold storage.

Various strategies have been used to ameliorate CI and to maintain flavor quality of peach fruit, including low-temperature conditioning (LTC) (2), hot air treatment (13), controlled atmosphere (14), 1-methylcyclopropene (1-MCP) treatment (15) and nitric oxide ultrasound treatment (11). Essential oils (EOs) are secondary metabolites directly extracted from aromatic and medicinal plants, with a natural or avirulent image, and exhibit remarkable antimicrobial, anti-pest and anti-oxidative properties due to their bioactive components. Accumulated evidence suggested that EO treatment effectively reduced postharvest diseases, maintained the overall quality, and extended the storage life of horticultural crops (16–19). Besides, EOs were also found to increase the resistance of tissues to decay through enhancing their antioxidant system. Chanjirakul et al. (20) showed that *Melaleuca alternifolia* EO promoted all of the tested antioxidant enzymes, including SOD, G-POD, AsA-POD, GSH-POD, GR, MDAR, and DHAR in raspberries. Thyme EO vapor was reported to reduce the incidence of brown rot in red and yellow skin peaches by increasing the antioxidant contents (catechin, chlorogenic, and caffeic acids) and the activities of defense-related enzymes (chitinase, β-1, 3-glucanase and phenylalanine ammonia-lyase), as well as enhancing antioxidant scavenging capacity (21). Moreover, EO treatment has also been reported to reduce chilling induced disorders of cold-sensitive fruits and vegetables such as lemon (22) and peach fruits (21). By analyzing the antioxidant content (including flavonoid, anthocyanin, and phenolic compounds), the antioxidant capacity (measured as oxygen radical absorbance capacity), and the activities of various antioxidant enzymes, Wang (23) demonstrated that EO which had beneficial effects on alleviating CI also were found to increase the antioxidant activity and free radical scavenging capacity in fruit tissues, implicating that high antioxidant enzyme activities may help in alleviating oxidative stress and in turn increase the resistance against CI. A correlation between higher antioxidant compounds and lower susceptibility to CI was also observed in peaches (24). Cinnamon essential oil (CEO) has been proven to be an excellent antioxidant and was widely applied in food industry, with its main compounds cinnamaldehyde, cinnamyl-acetate, eugenol, linalool, and camphor in the different varieties of cinnamon (25). Denkova-Kostova et al. (26) done the DPPH radical-scavenging assay, and CEO exhibited the highest antioxidant activity of four tested EOs. Recently, Yu et al. (27) showed that chitosan coatings with CEO reduced the peel browning by inhibiting the activity of PPO, the accumulation of MDA content by promoting POD activity, and the disease incidence by activating disease-resistant enzyme PAL in postharvest mangoes. Huang et al. (28) found that eugenol, a main component of CEO, could effectively retard the CI development in eggplant fruit by maintaining high total phenolics content associated with low activities of PPO and POD. To the best of our knowledge, very few studies have focused on the effect of CEO on CI symptom especially flavor quality of peach. Headspace-gas chromatography-ion mobility spectrometry (HS-GC-IMS) principally determined chemical ionic molecules based on the difference in the rate of migration of gas phase ions (29). In view of its advantages, such as no sample pretreatment, fast analysis time, low detection limits，HS-GC-IMS has been extensively applied in the food fields in recent years to assess volatile compounds with different origins or properties (30, 31).

Therefore, the objective of this study was to investigate the effect of CEO in vapor phase on the most prominent CI symptoms of cold-stored “Feicheng” peach, internal browning and aroma loss. Particularly, HS-GC-IMS was used to elucidate the changes in volatiles during refrigeration for the CEO treatment and the control. The results will provide new insight and reference for maintaining the aroma quality of cold-stored “Feicheng” peach.

## Materials and methods

### Fruit samples and treatments

Peach (*Prunuspersica* (L.) Batsch, cv. Feicheng) fruits were harvested at commercial maturity in Feicheng, Shandong, China, and were transferred to the laboratory within 6 h. Uniform fruits free of defects and mechanical damage were selected and randomly divided into three groups, the 0 d samples, stored at 0 ± 0.5°C, CEO-treated and stored at 0 ± 0.5°C. We previously assessed the dose–response effects of CEO on IB index of peaches, and the concentration of 50 μL/L for subsequent investigation have been selected (). To perform the CEO treatment, peach fruits were placed in a polypropylene container with 6.65 L air space. Subsequently, two pieces of sterilized filter paper were attached on the two sides of the inner surface with 332.5 μL of CEO added, then the lid was quickly covered and the final concentration of 50 μL/L of air (v/v) was obtained. The containers were sealed with PVC cling film and then stored at 0 ± 0.5°C for 28 d. Sampling points for IB measurement were set at 0, 7, 14, 21, 28 d. According to the IB results, we selected day 21 of cold storage to perform the examination of aroma content in peaches. Briefly, after cold storage for 21 d, the fruit were transferred for 2 d of shelf-life at 20°C for subsequent ripening. For clarity, these 2 d were indicated with “21 + 2”. Control fruits were subjected to the same condition without any CEO treatment. For each sampling point, 12 fruits were sampled with 4 fruits in each replicate. After determination of IB, all samples were immediately frozen in liquid nitrogen and stored at-80°C refrigerator for molecular analysis.

Pure-grade CEO (barks steam distillation; origin: China) was purchased from Guangzhou Hengxin Spice Co., Ltd., Guangzhou, China, and stored in the dark, at room temperature. The main composition of CEO was given in Table 1.

**Table 1:** Chemical compositions of cinnamon essential oil.

No.	Components	Retention time (s)	Percentage (%)
1	Camphorene	265.56	0.714
2	Benzaldehyde	274.62	7.907
3	4-isopropyltoluene	328.56	0.706
4	Limonene	332.94	0.175
5	Eucalyptol	336.36	0.031
6	Salicylaldehyde	347.82	0.612
7	Nonanal	398.28	0.350
8	2-carbitol	464.16	0.688
9	O-methoxybenzaldehyde	521.10	0.506
10	Phenethyl acetate	532.32	1.387
11	Trans-2-decenal	539.34	0.385
12	Trans-cinnamaldehyde	553.74	82.228
13	2,4-decadienal	587.52	3.174
14	Eugenol	617.10	0.301
15	2-undecenal	624.30	0.221
16	α-ylangene	733.14	0.228
17	O-methoxycinnamaldehyde	755.64	0.387

### IB index and color

Internal browning (IB) index was used to assess the degree of flesh browning by calculating the brown extent of each fruit after cutting peach along the axial diameter. IB index was measured based on the following formula of previous method (32): IB index = 100% × Σ [(internal browning scale) × (number of fruit at that internal browning scale)]/ [4 × total number of fruit in each treatment]. The internal browning scale was 0 = no browning; 1 = less than 1/4 browning; 2 = 1/4–1/2 browning; 3 = 1/2–3/4 browning; 4 = more than 3/4 browning.

CIELAB color space could provide parameters that are highly correlated with human visual perception of fruit color. Each color parameter is represented in a color diagram, where L* value ranges between 0 and 100, indicating the gradient from white to black; the positive a* value indicates a reddish-purple color, and negative a* value indicates a bluish-green color; the positive b* value indicates a yellow color, and negative b* value indicates a blue color (33). In this study, a portable NR10QC colorimeter (3nh Corp., China) was used to measure peach flesh browning. L* and a* values were measured on the flesh adhering firmly to the core (three points in each fruit). A total of nine peaches were randomly selected from the three replicates with three fruits of each group.

### HS-GC-IMS analysis

The volatile compounds analysis of peach fruits samples was implemented using a HS-GC-IMS instrument (Flavor Spec®, G. A. S., Department of Shandong HaiNeng Science Instrument Co., Ltd., Shandong, China). Two grams of the minced peach pulp (close to the skin, *ca.* 0.5 cm thickness) was transferred to a 20 mL headspace bottle and incubated at 40°C for 15 min. Then, 500 μL of headspace gas was sampled and automatically injected into a heated syringe at 45°C. GC was performed with a 15 m capillary column (FS-SE-54-CB-1, inner diameter: 0.35 mm) to separate the volatile compounds and coupled to IMS at 45°C. Nitrogen (99.999% purity) was used as the carrier gas at the following programmed flow rates: 2 mL/min for 2 min, 100 mL/min for 18 min, and then the flow stopped. The analytes were eluted, separated at 60°C in the column, and then ionized in an IMS ionization chamber at 45°C. A flow rate of 150 mL/min was used as the drift gas for IMS. All analyses were performed in triplicate.

GC-IMS data were analyzed using Laboratory Analytical Viewer (LAV), GC-IMS Library Search, the Reporter, Gallery plot and dynamic PCA plug-ins. LAV was used to view the analysis spectrum where each point represents a volatile compound. The GC-IMS Library Search used the IMS database to qualitatively analyze the substances. The Reporter plug-in could directly compare the difference between samples using 3D topographic plots. The Gallery plot plug-in could intuitively and quantitatively compare each volatile compound between different samples by fingerprint comparison. The dynamic PCA plug-in could cluster samples and determine the unknown substances using dynamic principal component analysis.

### Sensory evaluation

The potential effect of CEO on peach fruit aroma was analyzed in a panel test following the method of Duan et al. (34), with some modification. Peach flesh was cut into slices and placed in labeled glass dishes with lids. The interval between cutting fruit and sensory test was less than 30 min. It was presented the 21 + 2 d-CEO treated sample and 21 + 2 d-control fruit with an alternated order. After smelling, panelists were asked to indicate which one had more peach odor, and to evaluate their preference and acceptance.

### RNA extraction and quantification by real-time PCR

Trizol reagent (Invitrogen) was used for RNA isolation. RNA was converted to cDNA using PrimeScript™ RT reagent Kit with gDNA Eraser (Takara, Dalian, China) following the manufacturer’s instructions. Real-time PCR was conducted with CFX96™ Real-Time System (Bio-Rad, Hercules, United States) using TB Green Premix Ex Taq (TliRNaseH Plus) (Takara, Dalian, China). The fold change of the expression of the mRNA was calculated by the 2^-ΔΔCt^ method. Primers were listed in Table 2. The temperature program for qPCR was as follows: 94°C for 3 min, 45 cycles of 94°C for 5 s and 60°C for 30 s, followed by a final melting curve step from 65°C to 95°C.

**Table 2:** Oligomeric nucleotide primer sequences for quantitative reverse transcriptase polymerase chain reaction.

Gene	Sequence
*PpLOX1* forward *PpLOX1* reverse	5′- GTGGACTCACTGGGAGAGGA-3′ 5′- GTTGCACGACCATTTCACAC-3’
*PpLOX2* forward *PpLOX2* reverse	5’- TCACTACGACAAGCGGAACG-3′ 5′- GGTAGGACGGTTTGGCACAT-3’
*PpHPL1* forward *PpHPL2* reverse	5’- ACAAAATGCTTAGTTGGTGCTG-3′ 5′- CAATCTTGACAGTGGGGAGG-3’
*PpADH1* forward *PpADH1* reverse	5’- AACGCCCGACTAGTTTGTTG-3′ 5′- CGATCATTCTTCGGCAAATC-3′
*PpACTIN* forward *PpACTIN* reverse	5′- ACCTTCCAGCAGATGTGGATT-3′ 5′- CTGACCCCACCTCAACACAT-3’

### Statistical analysis

All data were expressed as mean value ± standard deviation (SD). Data were analyzed by one-way ANOVA and Duncan’s multiple range tests using SPSS 16.0 software. *p* value <0.05 was considered statistically significant.

## Results

### Effect of CEO vapor on internal browning during cold storage of “Feicheng” peach fruit

**Figure 1:**
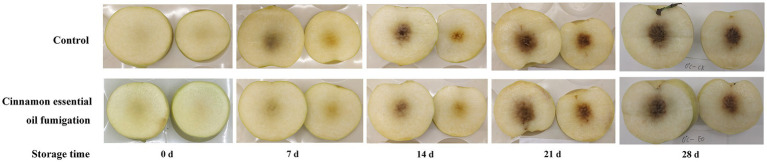
Morphological images of internal browning in “Feicheng” peach during cold storage.

As shown in Figure 1, browning from the core toward the flesh was evident in both control and CEO treatment from 7 d onwards. With the extension of cold storage, the browning color became deeper while the browning scale became larger. Especially during the period 14–21 d, the browning extent of the peach fruit flesh increased sharply. However, fruits treated with CEO exhibited less internal browning than the control, especially at 7, 14, and 21 d, and the difference between them was visually significant.

**Figure 2:**
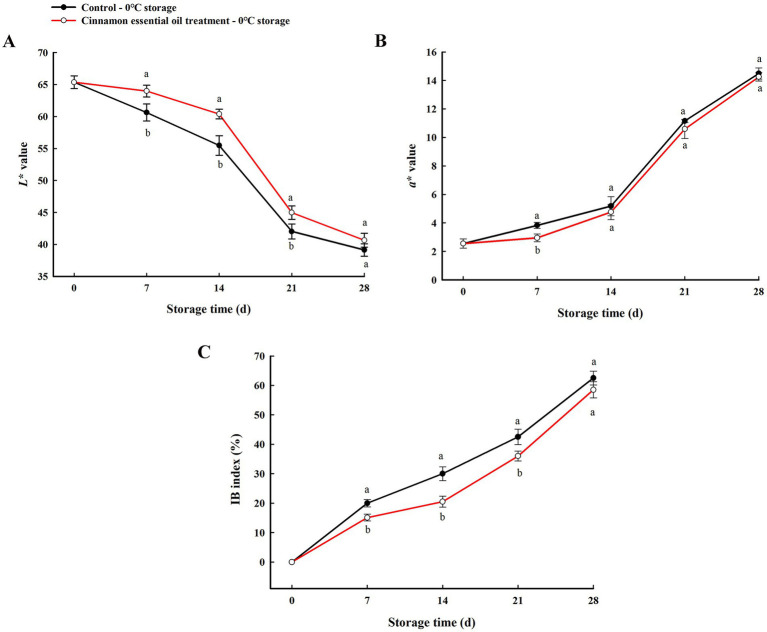
Changes of L* value **(A)**, a* value **(B)**, and IB index **(C)** of “Feicheng” peach section during cold storage. All the values are expressed as means ± SD of three replicates. The different normal letters indicate significant differences at 0.05 level (Duncan’s test) and the same letter means no significant difference between Control and CEO-treated groups.

Furthermore, lightness (L* value) and chrome a* value, combined IB index were used to quantitatively assess the degree of browning of the internal tissues of “Feicheng” peach fruit during cold storage. Previous studies have reported a high correlation between the L* parameter and peach flesh browning, with decreasing L* values being a reliable indicator of browning (35, 36). a* parameter was frequently used to assess fresh-cut products, as reported in fresh apple or pear slices, and it was considered that a* value of the pulp was the best indicator of browning (37, 38). In our study, the changes in L* and a* values of the flesh around the core may indicate browning (dark and red flesh) because before storage flesh was white. The L* value tended to decrease with increasing refrigeration time, while a* value increased significantly, indicating that the low temperature stress darkened the internal browning tissue to a more intense red color (Figures 2A,B). At the same time, higher IB index was observed throughout experimental period (Figure 2C). Compared with the control, the internal flesh color of the fruit treated with CEO was much lighter (L* value increased), less chromatic (a* value decreased), and the internal browning scale showed slight decline. The differences of L* value at 7, 14, 21 d, a* value at 7 d, and IB index at 7, 14, 21 d, between the CEO-treated and the control fruit were significant (*p* < 0.05), of which the parameters L* value and IB index were highly correlated with the above visual evaluation. These data revealed that CEO treatment was effective in alleviating the severity and delaying the development of internal browning of “Feicheng” peach fruit in early stage of cold storage.

### Effect of CEO vapor on aroma-related volatiles content after cold storage of “Feicheng” peach fruit

**Figure 3:**
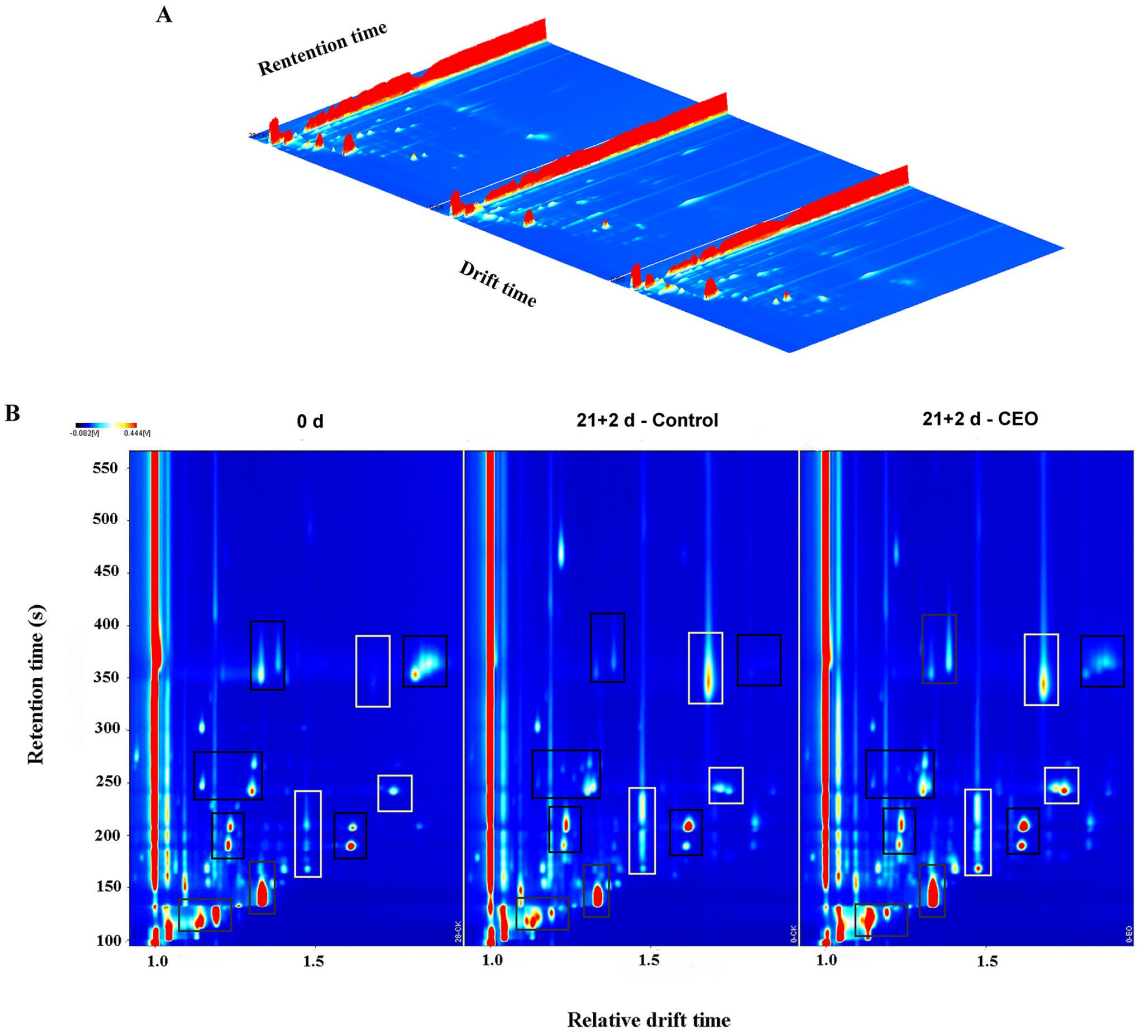
HS-GC-IMS 3D **(A)** and 2D **(B)**-topographic plots of volatile compounds in “Feicheng” peach pulp from different treated groups. In the areas framed by black boxes, the content of volatile compounds decreased or disappeared after cold storage, and in the areas framed by white boxes, the content increased or new volatiles appeared.

The data for volatile compounds in different treated groups analyzed by HS-GC-IMS were presented by 3D topography in Figure 3A, where the *X*-axis represented the ion migration time for identification, the *Y*-axis represented the retention time of the gas chromatograph, and the *Z*-axis represented the peak height used for quantification. As shown in Figure 3A, we found that the volatiles composition in each treatment group were very similar, but the signal intensities were slightly different.

By normalizing the time of ion migration and the position of the reactive ion peak (RIP), we obtained the top view of GC-IMS topographic plot of volatile compounds in peach fruits with different treatments (Figure 3B). The whole spectrum represents the headspace composition of the sample. It could be seen that the majority of the signals was located in the retention time of 100–400 s and the drift time of 1.0–1.8. Signal intensity was represented by the color. White indicated lower intensity and red indicated higher intensity. The darker the color was, the greater the intensity was. Compared with the 0 d samples, the red spot areas framed by black boxes at the retention time range of 110–130 s, 130–170 s, 180–210 s, 240–270 s, and 340–380 s were distinctly smaller or even disappeared in the control samples stored after 21 d of cold storage plus 2 d of shelf life, showing that the signals of volatile substance were much lower than those of the day 0 samples. However, almost all of the above weakened spots or areas were strengthened in CEO-treated samples under the same storage conditions. Moreover, the signals framed by white boxes for the two storage groups located in the retention time range of 160–230 s, 240–260 s, and 330–380 s were stronger than those observed in the 0 d samples. Among them, it was noteworthy that only the CEO-treated samples exhibited higher signal intensity during the retention time of 240–260 s. After long-term cold storage, the signals of characteristic aroma-related volatiles disappeared or their intensity decreased. In contrast, CEO treatment effectively prevented the loss of these volatiles, leading to a certain increase in these signal intensities.

The fingerprinting technique was used to quantitatively reveal the dynamic changes of each volatile compound. The appreciated visual signals were listed, respectively, by Gallery plot for intuitive comparison. Therefore, variance between different treatments were evident. Due to the formation of adducts between the analyzed ions and neutral molecules when passing through the drift tube, some single compounds were observed more than one signal (e.g., dimers or trimers) in the drift time. The results demonstrate that the formation of dimers or high clusters associated with compounds having high proton affinity and/or higher concentration in the analyte.

**Figure 4:**
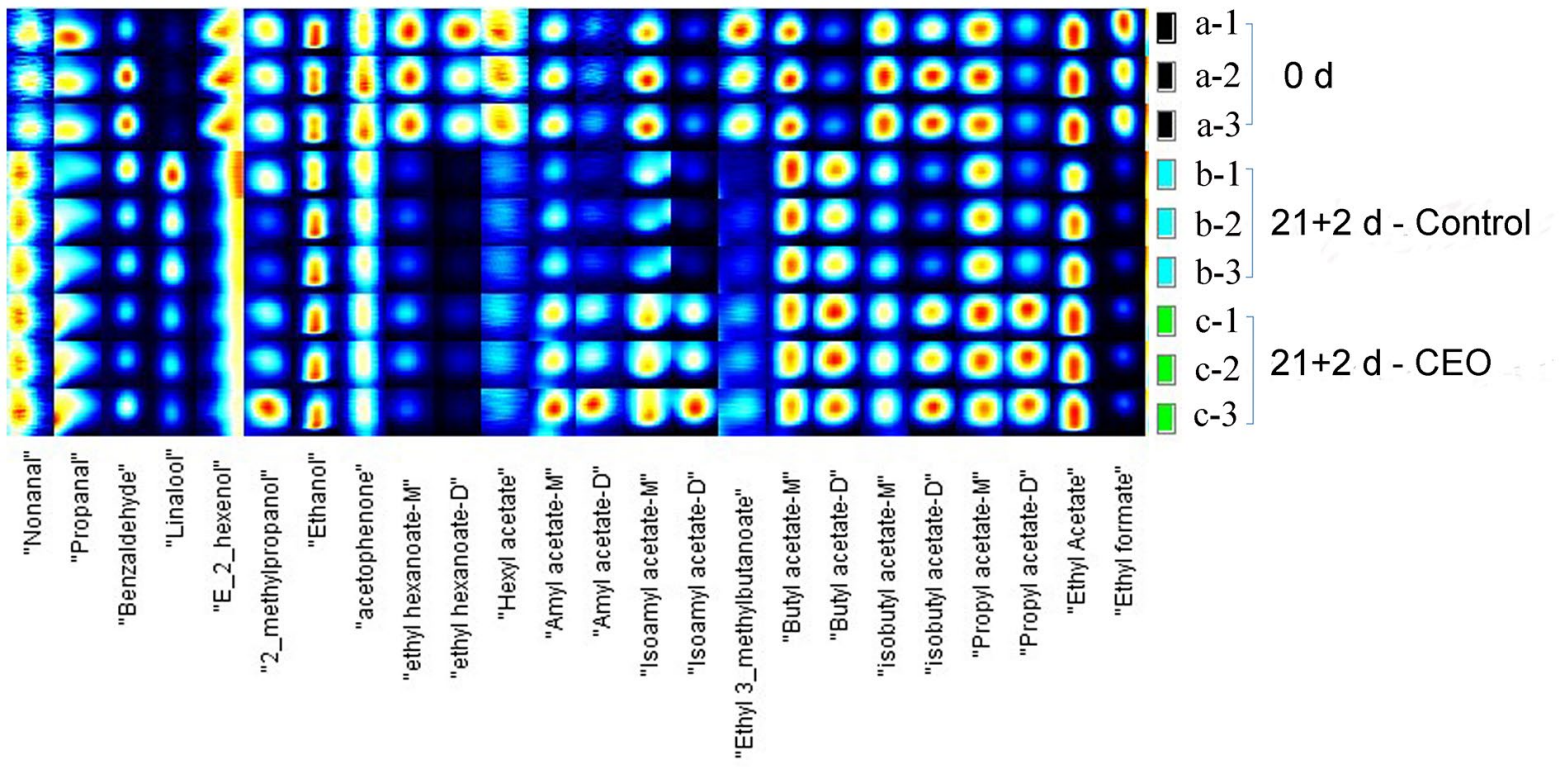
Fingerprint spectra of volatile compounds in “Feicheng” peach pulp from different treated groups.

A total of 24 typical target compounds from topographic plots were identified by the GC-IMS Library, including ester, alcohol, aldehyde, and ketone, based on retention and drift times (Figure 4; Table 3). As presented, the signal intensities of propanal, benzaldehyde, (E)-2-hexenol, 2-methylpropanol, acetophenone, hexyl acetate, amyl acetate, isoamyl acetate, isobutyl acetate, propyl acetate, and ethyl acetate in the 0 d samples were much higher than those in the control samples after 21 d of storage plus 2 d of shelf life (*p* < 0.05), whereas ethyl hexanoate, ethyl 3-methylbutanoate, and ethyl formate were not detected in the latter samples. Among those weaker or lost signals, improved signal intensities of propanal, 2-methylpropanol, ethyl hexanoate, hexyl acetate, amyl acetate, isoamyl acetate, isobutyl acetate, propyl acetate, ethyl acetate and ethyl formate were seen in CEO-treated samples (*p* < 0.05), suggesting that the declined concentration level of these compounds were partly recovered after CEO fumigation.

**Table 3:** Peak areas of volatile compounds of “Feicheng” peach from different treatment groups.

Compound	RI^1^	Rt [sec]^2^	Dt^3^	Peak areas of peach samples		
				0 d	21 + 2 d Control	21 + 2 d CEO
Esters (16)						
Ethyl hexanoate Monomer	1012.4	353.362	1.3352	624.05 ± 17.00^a^	158.21 ± 21.76^c^	241.22 ± 6.27^b^
Ethyl hexanoate Dimer	1013.0	354.095	1.8168	603.40 ± 3.65^a^	64.13 ± 4.52^c^	138.58 ± 4.45^b^
Hexyl acetate	1009.0	349.316	1.4124	107.40 ± 9.47^a^	33.11 ± 0.92^c^	39.10 ± 3.59^b^
Amyl acetate Monomer	920.9	268.66	1.3124	235.09 ± 2.07^a^	149.99 ± 13.11^c^	251.89 ± 3.54^b^
Amyl acetate Dimer	920.0	268.063	1.7632	34.05 ± 3.71^b^	27.68 ± 0.87^c^	51.24 ± 0.80^a^
Isoamyl acetate Monomer	878.4	242.373	1.3052	573.40 ± 0.45^b^	398.43 ± 0.65^c^	659.87 ± 4.86^a^
Isoamyl acetate Dimer	878.4	242.373	1.7472	294.74 ± 0.94^b^	153.22 ± 0.34^c^	750.76 ± 3.95^a^
Ethyl 3-methylbutanoate	850.8	227.436	1.2573	61.78 ± 3.04^a^	16.27 ± 3.65^c^	27.72 ± 3.13^b^
Butyl acetate Monomer	811.7	208.318	1.2399	522.86 ± 1.63^c^	653.86 ± 2.78^a^	644.54 ± 3.17^b^
Butyl acetate Dimer	810.4	207.721	1.6197	333.10 ± 4.89^c^	959.99 ± 6.55^b^	1334.64 ± 7.39^a^
Propyl acetate Monomer	715.1	167.347	1.1663	223.36 ± 15.47^ab^	214.17 ± 3.69^b^	246.77 ± 12.49^a^
Propyl acetate Dimer	717.2	168.126	1.4788	142.14 ± 11.31^c^	168.43 ± 0.45^b^	392.11 ± 8.28^a^
Isobutyl acetate Dimer	770.9	189.938	1.6143	760.52 ± 8.08^a^	230.58 ± 10.58^c^	411.67 ± 3.30^b^
Isobutyl acetate Monomer	770.3	189.679	1.2307	594.96 ± 1.60^b^	333.30 ± 19.96^c^	665.26 ± 2.85^a^
Ethyl acetate	616.5	139.042	1.3353	6769.44 ± 47.21^b^	6156.20 ± 31.35^c^	7605.81 ± 49.97^a^
Ethyl formate	535.6	120.345	1.1931	3240.89 ± 14.68^a^	642.49 ± 25.80^c^	838.21 ± 9.38^b^
Alcohols (4)						
Linalool	1092.1	465.818	1.2255	105.53 ± 6.79^c^	171.48 ± 4.13^b^	317.03 ± 2.87^a^
(E)-2-hexenol	853.0	228.631	1.1805	153.56 ± 7.22^a^	121.45 ± 3.73^b^	109.29 ± 4.57^c^
2-methylpropanol	674.7	154.103	1.3688	191.80 ± 13.92^a^	77.88 ± 0.90^c^	142.08 ± 1.00^b^
Ethanol	466.8	104.505	1.0483	3642.83 ± 45.52^b^	3932.98 ± 27.79^a^	3958.15 ± 48.91^a^
Aldehydes (3)						
Nonanal	1108.8	490.999	1.4865	135.68 ± 18.21^c^	171.48 ± 4.13^a^	166.04 ± 5.15^b^
Benzaldehyde	965.1	303.312	1.1486	309.03 ± 10.04^a^	202.05 ± 28.55^b^	155.87 ± 13.08^c^
Propanal	523.2	117.489	1.1462	1268.95 ± 4.23^b^	1181.22 ± 2.91^c^	1339.09 ± 6.85^a^
Ketones (1)						
Acetophenone	1056.9	413.336	1.1904	277.29 ± 21.25^a^	201.81 ± 5.82^c^	224.70 ± 6.05^b^

Different letters on the same line represent differences between different treatment samples, *p* < 0.05.

1 Represents the retention index calculated using n-ketones C4-C9 as external standard on FS-SE-54-CB-1 column.

2 Represents the retention time in the capillary GC column.

3 Represents the drift time in the drift tube.

On the other hand, some volatile compounds including nonanal, linalool, and butyl acetate were distinctive and had higher concentrations in the two storage groups than that on harvest day (*p* < 0.05). There was only one compound, propyl acetate, detected a remarkable promotion of signal intensity in CEO-treated samples, whereas almost undetectable in both samples at day 0 and the control at day 21 + 2. Propyl acetate, frequently described as having “fruity” and “sweet” odor attributes, was identified as a major compound that contributed to aroma formation in strawberry and apple fruits (39, 40), but it was rarely detected in peach fruits. Furthermore, CEO-treated samples had higher concentrations of amyl acetate, isoamyl acetate, and butyl acetate than those in the two other groups (*p* < 0.05). The above 4 esters, both newly formed or quantitatively increased, resulted in a richer and more diverse peach fruit flavor perception. Thus, the CEO treatment effectively improved the refrigerated aroma quality of “Feicheng” peach.

### Similarity analysis of fingerprint based on PCA

**Figure 5:**
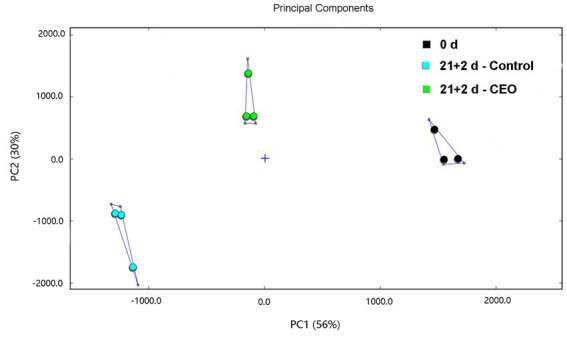
PCA score plot based on the signal intensity obtained with different treated samples.

Principal component analysis (PCA) was performed to highlight the differences in volatile profiles of peach fruits based on signal intensities. PCA of volatile compounds in different treated-peach fruit was shown in Figure 5. It presented the first two principal components that described 56 and 30% of the accumulative variance contribution rate. The three treatment groups were remarkably distinguished in the distribution map. Fruits samples for 0 d were mainly located in the positive areas of PC1 and PC2. Peach fruits of the control at day 21 + 2 were absolutely located in the negative areas of PC1 and PC2, while peach fruits of CEO-treated samples were mainly located on the negative area of PC1 and the positive area of PC2. Except ethanol, all of the content of volatile compounds listed in Table 3 were significantly different among treatment groups (*p* < 0.05). These exhibited major changes of aroma volatiles in control after 21 d of cold storage plus 2 d of shelf life compared with 0 d peach and obvious recovery in CEO-treated fruits.

### Sensory evaluation

**Figure 6:**
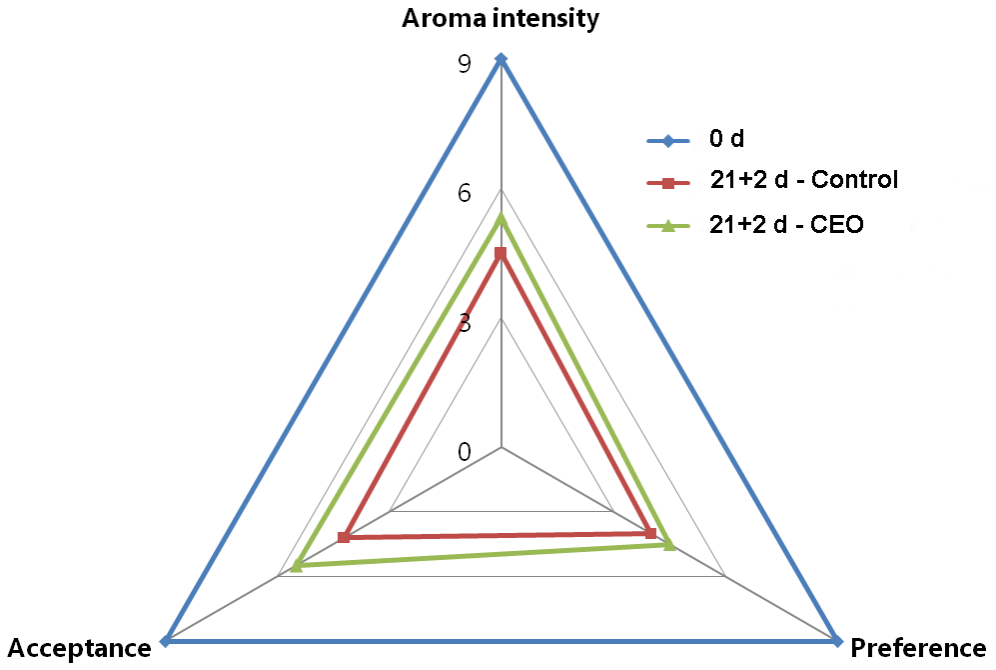
Sensory evaluation of “Feicheng” peach fruit with different treatments (*n* = 10 panelists).

The aroma profiles of peach fruits with different treatment were perceived by a sensory panel consisting of 10 trained individuals. The panelists recognized that CEO treated fruits had more peach aroma intensity than the control after 21 d of cold storage plus 2 d of shelf life, with more preference and better acceptance (Figure 6), which further suggested that CEO treatment could effectively alleviate the occurrence of aroma-related volatiles loss and promoted their recovery in peach fruit during long-term cold storage.

### Effect of CEO vapor on expression of genes related to volatiles synthesis during cold storage of “Feicheng” peach fruit

**Figure 7:**
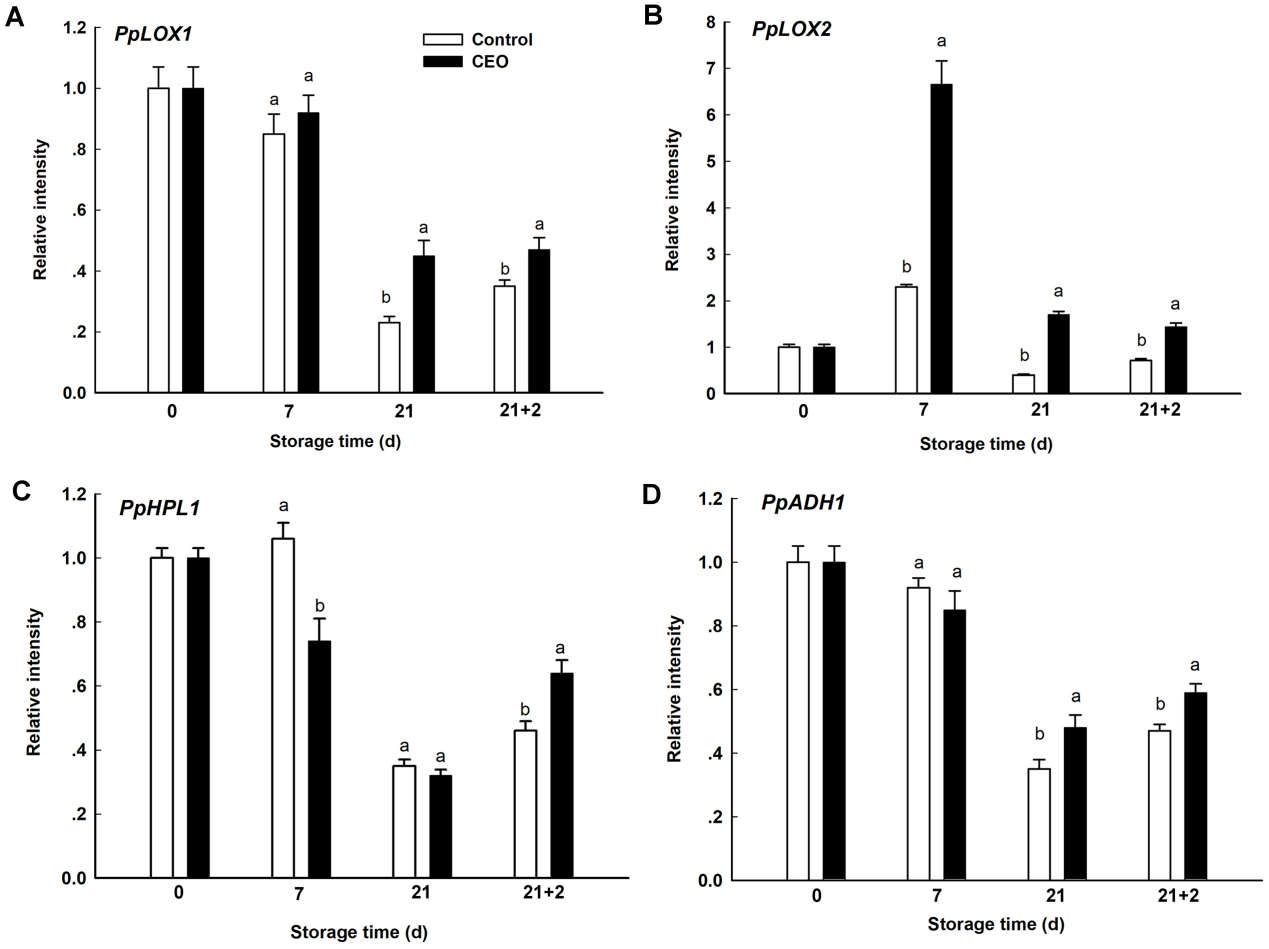
Expression of genes related to volatiles synthesis during cold storage plus shelf life. **(A)** PpLOX1, **(B)** PpLOX2, **(C)** PpHPL1, **(D)** PpADH1. 21 + 2 represents the second day during shelf life. All the values are expressed as means ± SD of three replicates. The different normal letters indicate significant differences at 0.05 level (Duncan’s test) and the same letter means no significant difference between Control and CEO-treated groups.

For further understanding the molecular mechanism of the effect of CEO on peach fruit volatiles, changes in transcript level of genes involved in LOX pathway were determined. The transcript level of *PpLOX1* tended to successive decline over cold storage period, followed by an obvious elevation when comparing the level at 21 d with that during 2 d of shelf life in both of two groups. *PpLOX1* in CEO-treated group displayed higher expression at 21 d and 21 + 2 d (*p* < 0.05; Figure 7A). However, *PpLOX2* showed different response patterns. *PpLOX2* in both two groups showed sharp increase at the very beginning of cold storage, subsequently decreased till the end of shelf life, and CEO treatment significantly promoted its expression from 7 d to 21 + 2 d (*p* < 0.05; Figure 7B). During cold storage, the transcript level of *PpHPL1* was generally decreased, with a burst after transferring to 20°C. Significant higher level was found in control at 7 d, while CEO induced its expression after 2 d of shelf life (*p* < 0.05; Figure 7C). Similar expression trend was also observed for *PpADH1* throughout the experimental period, but no significant difference was observed between CEO treatment and the control, while CEO induced its expression since 21 d (*p* < 0.05; Figure 7D).

## Discussion

Cold injury involves a series of cellular stress-responses and crosstalk with ripening and senescence processes, at the biochemical, physiological, and molecular levels (41). Peach is more sensitive to low temperature, and the reduction of peach fruit quality after cold storage is mainly caused by the development of chilling injury (1). Internal flesh browning is due to metabolic disorders (such as low temperature-induced ATP deficiency, manifesting in discoloration of the fruit flesh, turning the yellowish or white flesh brown), which is one of the most typical symptoms of CI (42). Here, significant IB in “Feicheng” peach fruits was observed after 7 d cold storage of 0°C (Figure 1), and this result was inconsistent with previous studies which noticed that IB was not visible until 21 d at low temperature (2, 11). It has been reported that CI visual symptoms especially internal browning, developed faster and more intensely when susceptible fruits were stored at 0°C than those stored at temperatures between 3–5°C (43), and were more frequently observed in white flesh peach cultivars (1). In our study, “Feicheng” peach, a white flesh cultivar., was selected and stored at the temperature of 0°C. Cai kept the storage temperature at 4°C, while Wang stored “Hujingmilu” peach, a naturally deeply colored cultivar at 0°C (2, 11). The discrepancy suggested distinct susceptibility to low temperature among different cultivars or storage temperature of peach fruits. Besides, the maturity at which peaches were harvested greatly influenced their CI symptoms and ultimate flavor (44).

Loss of aroma is the earliest symptom of CI, but it is also a particularly insidious damage symptom when CI occurs. For peaches, CI reduces the expression of key genes involved in aromatic ester formation and disrupts the balance of taste and aroma volatiles, which in turn negatively impact fruit flavor and consumers’ eating experiences (45). Esters are the most abundant volatile compounds in peach fruits, and they play a critical role in volatile aroma produced during ripening (46). In this study, the main esters were ethyl acetate, ethyl formate, isobutyl acetate, and ethyl hexanoate. Besides the volatile esters, ethanol and propanal are characteristic components of “Feicheng” peach aroma (Table 3). Impaired flavor quality after cold storage correlated with lowered aroma-related volatiles, particularly for fruity note esters such as hexyl acetate, (E)-2-hexenyl acetate, and (Z)-3-hexenyl acetate (47). Similarly, difference in volatile profiles of peach fruits after long-term refrigeration was revealed by HS-GC-IMS analysis. Most of volatiles decreased significantly when cold storage was extended to 21 d (Figure 4).

The effectiveness of CEO in alleviating CI was also found in “Jim Dandy” peach (21) and other cold-sensitive fruit such as Lemon fruit (22), which were mainly focus on flesh browning and texture disorders. Although EO treatment could increase cold tolerance and reduce CI in multiple fruit species, the effect of CEO on flavor quality of peach fruit after cold storage needs to be investigated. Our dataset in peach fruit showed that CEO treatment promoted the recovery of fruity note volatile esters than control during shelf-life after cold storage. These fruity esters are mainly consisted of hexyl acetate, ethyl hexanoate, isoamyl acetate, and ethyl formate (Figure 4), which are positively associated with consumer liking and are derived from the LOX-mediated fatty acid oxidation pathway (48, 49). Moreover, the levels of nonanal, linalool, and butyl acetate were all lower than that on harvest day, regardless of CEO-treated or control fruits (Figure 4), which could be due to the normal production and release of these compounds during the postharvest biochemical process of peach fruits, or a ripening hindrance sensitive to low temperatures leading to a forced transfer of certain substances metabolic pathways. Therefore, it is interesting to examine the mechanism in future. The key factor influencing the consumer’s choice for peaches is sensory quality, so it is vital to conduct sensory quality evaluation and consumer satisfaction test (50). Our sensory analyses demonstrated that CEO vapor treatment is effective because it not only delays the development of IB but also recovers the emission of aroma-related volatiles (Figure 6).

Fruit volatile compounds mainly produced from β-oxidation and LOX pathways, during which LOX, HPL and ADH are key enzymes. Gene family member of *PpLOX1*, *PpHPL1* and *PpADH1* correlated with volatile ester and lactone biosynthesis in ripening peach fruit (49, 51). Previous research reported that expression level of two LOX genes and one HPL gene highly correlated with the formation of (E)-2-hexenal and n-hexenal in peach (52). For banana fruit, LOX and HPL were reported to play an important role in volatile ester production (53). Similar results were also found in apple fruit under cold stress, where LOX was a key control point for successful recovery of fruit ability for volatile ester production (54). Here, decreased expression patterns were observed for *PpLOX1*, *PpHPL1,* and *PpADH1* with extended cold storage time, while *PpLOX2* showed a sharp rise at the very beginning of refrigeration in both two groups. After transferring peach from 0°C to 20°C, the four genes all showed significant higher level in CEO-treated peach fruit than controls at 21 + 2 d (Figure 7). Considering the higher emission of aroma-related volatiles in peach treated with CEO, which may be concomitant with the elevated levels of these four transcripts. Similarly, the decrease of LOX activity possibly led to shortage of lipid precursors for ester biosynthesis in pear fruit (55). Fatty acids (FAs) were important precursors responsible for the biosynthesis of volatile compounds. Besides, FAs composition correlated with membrane stability in response to abiotic stresses and was regulated by LOX and FADs (fatty acid desaturases) (56). Evidence suggested that the co-regulation of LOX and FADs was a crucial loop linked the state of unsaturated fatty acids and jasmonate accumulation to enhance cold tolerance in plants (2). Previous studies implied that the biosynthesis of volatile compounds also could be tightly modified by FADs in peach fruits (11). Therefore, how CEO could affect FADs and other genes involved in the lipid biosynthetic pathway in peach fruits under cold, and the systematic association of all these factors affecting chilling sensitivity should be considered for further validation. Furthermore, much work is required to provide a comprehensive interaction between promotion of aroma-related volatiles by CEO treatment and transcriptional profiles, and to obtain new insights underlying postharvest molecular biology, including the protection of peach fruit under cold stress.

In addition, it should be noted that although EOs have been promising eco-friendly and effective food preservatives, their inherent strong odors might alter the original and typical flavor/taste of concerned food products and adversely affect their organoleptic properties, which to some extent limits the application of EOs (57–59). As expected, compared with the chemical compositions of CEO listed in Table 1, no other characteristic compounds were found in the CEO-treated peach fruits except for benzaldehyde and nonanal that were also intrinsic aroma components of “Feicheng” peach. So, in this study “off-odors” were completely absent in peach pulp, in contrast, the CEO vapors helped to maintain the peach aroma during cold storage.

## Conclusion

In conclusion, CEO vapor enhanced chilling tolerance of “Feicheng” peaches. CEO treatment reduced the severity of internal browning and delayed the onset of browning in “Feicheng” peaches at the early stage of cold storage. In addition, CEO not only promoted the recovery of some of the major volatiles that had declined or been lost in peaches suffered from CI, but also stimulated the release of more beneficial aroma-related volatiles, which made the flavor perception richer and more diverse. The volatile-related improvement on peach fruit was also supported by the up-regulated *PpLOX1*, *PpLOX2*, *PpHPL1,* and *PpADH1*. These results together suggested that CEO vapor treatment could be an effective way to reverse the reduction of aroma and improve the flavor quality of cold-stored “Feicheng” peach. In future, biochemical and molecular mechanisms of CI alleviation by CEO need to be further investigated.

## Data availability statement

The original contributions presented in the study are included in the article/Supplementary material, further inquiries can be directed to the corresponding author.

## Author contributions

DW: methodology, investigation, writing-original draft, and visualization. JZ: investigation and formal analysis. W-yC: resources. HZ: conceptualization, supervision, project administration, and funding acquisition. YJ: methodology, and writing–review and editing. All authors contributed to the article and approved the submitted version.

## Funding

This study was supported by the Key Research and Development Program of Shandong Province (2021TZXD01304).

## Conflict of interest

The authors declare that the research was conducted in the absence of any commercial or financial relationships that could be construed as a potential conflict of interest.

## Publisher’s note

All claims expressed in this article are solely those of the authors and do not necessarily represent those of their affiliated organizations, or those of the publisher, the editors and the reviewers. Any product that may be evaluated in this article, or claim that may be made by its manufacturer, is not guaranteed or endorsed by the publisher.

## References

[1] Lurie S Crisosto CH. Chilling injury in peach and nectarine. Postharvest Biol Technol. (2005, 2005) 37:195–8. doi: 10.1016/j.postharvbio.2005.04.012

[2] Wang K Yin X Zhang B Grierson D Xu CJ. Transcriptomic and metabolic analyses provide new insights into chilling injury in peach fruit. Plant Cell Environ. (2017) 40:1531–51. doi: 10.1111/pce.12951, PMID: 28337785

[3] Yin XC Ji SJ Cheng SC Zhou Q Zhou X Luo ML. Methyl jasmonate alleviates the reduced release of aroma-related esters in ‘Nanguo’ pears by regulating ethylene biosynthesis and signal transduction. Int J Food Sci Technol. (2021) 56:814–4. doi: 10.1111/ijfs.14725

[4] Aubert C Gunata Z Ambid C Baumes R. Changes in physicochemical characteristics and volatile constituents of yellow and white-fleshed nectarines during maturation and artificial ripening. J Agric Food Chem. (2003) 51:3083–91. doi: 10.1021/jf026153i, PMID: 12720396

[5] Niu YW Deng JM Xiao ZB Zhu JC. Characterization of the major aroma-active compounds in peach (*Prunus persica* L. Batsch) by gas chromatography-olfactometry, flame photometric detection and molecular sensory science approaches. Food Theatr Res Int. (2021) 147:110457. doi: 10.1016/j.foodres.2021.110457 34399457

[6] Wang YJ Yang CX Li SH Yang L Wang YN Zhao LB. Volatile characteristics of 50 peaches and nectarines evaluated by HP–SPME with GC–MS. Food Chem. (2009) 116:356–4. doi: 10.1016/j.foodchem.2009.02.004

[7] Bianchi T Weesepoel Y Koot A Iglesias I Eduardo I Gratacós-Cubarsí M. Investigation of the aroma of commercial peach (*Prunus persica* L. Batsch) types by proton transfer reaction-mass spectrometry (PTR-MS) and sensory analysis. Food Res Int. (2017) 99:133–6. doi: 10.1016/j.foodres.2017.05.007, PMID: 28784469

[8] Jia HJ Araki A Okamoto G. Influence of fruit bagging on aroma volatiles and skin coloration of ‘Hakuho’ peach (*Prunus persica* Batsch). Postharvest Biol Technol. (2005) 35:61–8. doi: 10.1016/j.postharvbio.2004.06.004

[9] Cai HF An XJ Han S Jiang L Yu ML. Effect of 1-MCP on the production of volatiles and biosynthesis-related gene expression in peach fruit during cold storage. Postharvest Biol Technol. (2018) 141:50–7. doi: 10.1016/j.postharvbio.2018.03.003

[10] Lavilla T Recasens I Lopez ML. Production of volatile aromatic compounds in big top nectarines and royal glory peaches during maturity. Acta Hortic. (2001) 553:233–4. doi: 10.17660/ACTAHORTIC.2001.553.51

[11] Cai HF Han S Yu ML Ma RJ Yu ZF. Exogenous nitric oxide fumigation promoted the emission of volatile organic compounds in peach fruit during shelf life after long-term cold storage. Food Res Int. (2020) 133:109135. doi: 10.1016/j.foodres.2020.109135, PMID: 32466940

[12] Zhao YY Song CC Brummell DA Qi SN Lin Q Bi JF. Salicylic acid treatment mitigates chilling injury in peach fruit by regulation of sucrose metabolism and soluble sugar content. Food Chem. (2021) 358:129867. doi: 10.1016/j.foodchem.2021.129867, PMID: 33979685

[13] Zhou DD Sun Y Li MY Zhu T Tu K. Postharvest hot air and UV-C treatments enhance aroma-related volatiles by simulating the lipoxygenase pathway in peaches during cold storage. Food Chem. (2019) 292:294–3. doi: 10.1016/j.foodchem.2019.04.049, PMID: 31054678

[14] Infante R Meneses C Crisosto CH. Preconditioning treatment maintains taste characteristic perception of ripe ‘September Sun’ peach following cold storage. Int J Food Sci Technol. (2009) 44:1011–6. doi: 10.1111/j.1365-2621.2008.01864.x

[15] Wang Q Wei YY Jiang S Wang XX Xu F Wang HF. Flavor development in peach fruit treated with 1-methylcyclopropene during shelf storage. Food Res Int. (2020) 137:109653. doi: 10.1016/j.foodres.2020.109653, PMID: 33233232

[16] He JL Wu DT Zhang Q Chen H Li HY Han QH. Efficacy and mechanism of cinnamon essential oil on inhibition of Colletotrichum acutatum isolated from ‘Hongyang’ kiwifruit. Front Microbiol. (2018) 9:1288. doi: 10.3389/fmicb.2018.01288, PMID: 29967599 PMC6015887

[17] Ju J Xu XM Xie YF Guo YH Cheng YL Qian H. Inhibitory effects of cinnamon and clove essential oils on mold growth on baked foods. Food Chem. 240:850–5. doi: 10.1016/j.foodchem.2017.07.120, PMID: 28946351

[18] Sivakumar D Bautista-Baños S. A review on the use of essential oils for postharvest decay control and maintenance of fruit quality during storage. Crop Prot. (2014) 64:27–37. doi: 10.1016/j.cropro.2014.05.012

[19] Xu JY Shao XF Wei Y Xu F Wang HF. iTRAQ proteomic analysis reveals that metabolic pathways involving energy metabolism are affected by tea tree oil in Botrytis cinerea. Front Microbiol. (2017) 8:1989. doi: 10.3389/fmicb.2017.01989, PMID: 29075250 PMC5643485

[20] Chanjirakul K Wang SY Wang CY Siriphanich J. Effect of natural volatile compounds on antioxidant capacity and antioxidant enzymes in raspberries. Postharvest Biol Technol. (2006) 40:106–5. doi: 10.1016/j.postharvbio.2006.01.004

[21] Khumalo KN Tinyanea P Soundy P Romanazzi G Glowacz M. Effect of thyme oil vapor exposure on the brown rot infection, phenylalanine ammonia-lyase (PAL) activity, phenolic content and antioxidant activity in red and yellow skin peach cultivars. Sci Hortic. (2017) 214:195–9. doi: 10.1016/j.scienta.2016.11.044

[22] Obenland DM Margosan DA Houck LG Aung LH. Essential oils and chilling injury in lemon. HortScience. (1997) 32:108–1. doi: 10.21273/HORTSCI.32.1.108

[23] Wang CY. Reducing chilling injury and maintaining quality of horticultural crops with natural products and their derivatives. Acta Hortic. (2006) 712:285–12. doi: 10.17660/ActaHortic.2006.712.31

[24] Abidi W Cantín CM Jiménez S Giménez R Moreno MÁ Gogorcena Y. Influence of antioxidant compounds, total sugars and genetic background on the chilling injury susceptibility of a non-melting peach (*Prunus persica* (L.) Batsch) progeny. J Sci Food Agric. (2015) 95:351–8. doi: 10.1002/jsfa.6727, PMID: 24796322

[25] Cardoso-Ugarte GA López-Malo A Sosa-Morales ME (2016) Chapter 38-cinnamon (*Cinnamomum zeylanicum*) essential oils, editor(s): Victor R. Preedy, essential oils in food preservation, flavor and safety, New York: Academic Press, 339–347

[26] Denkova-Kostova R Teneva D Tomova T Goranov B Denkova Z Shopska V. Chemical composition, antioxidant and antimicrobial activity of essential oils from tangerine (*Citrus reticulata* L.), grapefruit (Citrus paradisi L.), lemon (Citrus lemon L.) and cinnamon (*Cinnamomum zeylanicum* Blume). Z Naturforsch C J Biosci. (2020) 76:175–5. doi: 10.1515/znc-2020-0126, PMID: 33909955

[27] Yu K Xu J Zhou L Zou L Liu W. Effect of chitosan coatings with cinnamon essential oil on postharvest quality of mangoes. Foods. (2021) 10:3003. doi: 10.3390/foods10123003, PMID: 34945553 PMC8700884

[28] Huang QH Qian XC Jiang TJ Zheng XL. Effect of eugenol fumigation treatment on chilling injury and CBF gene expression in eggplant fruit during cold storage. Food Chem. (2019) 292:143–12. doi: 10.1016/j.foodchem.2019.04.048, PMID: 31054659

[29] Shvartsburg A. Ion mobility spectrometry (IMS) and mass spectrometry (MS). Encycl Spectrosc Spectrom. (2010) 23:1140–8. doi: 10.1016/B978-0-12-374413-5.00012-9

[30] Gerhardt N Birkenmeier M Sanders D Rohn S Weller P. Resolution-optimized headspace gas chromatography-ion mobility spectrometry (HS-GC-IMS) for nontargeted olive oil profiling. Anal Bioanal Chem. (2017) 409:3933–42. doi: 10.1007/s00216-017-0338-2, PMID: 28417171

[31] Rodríguez-Maecker R Vyhmeister E Meisen S Rosales Martinez A Kuklya A Telgheder U. Identification of terpenes and essential oils by means of static headspace gas chromatography-ion mobility spectrometry. Anal Bioanal Chem. (2017) 409:6595–03. doi: 10.1007/s00216-017-0613-2, PMID: 28932891

[32] Wang K Shao XF Gong YF Zhu Y Wang HF. The metabolism of soluble carbohydrates related to chilling injury in peach fruit exposed to cold stress. Postharvest Biol Technol. (2013) 86:53–61. doi: 10.1016/j.postharvbio.2013.06.020

[33] McGuire RG. Reporting of objective color measurements. HortScience. (1992) 27:1254–5. doi: 10.21273/HORTSCI.27.12.1254

[34] Duan WY Yang C Cao XM Zhang C Liu HR Chen KS. Transcriptome and DNA methylome analysis reveal new insights into methyl jasmonate-alleviated chilling injury of peach fruit after cold storage. Postharvest Biol Technol. (2022) 189:111915. doi: 10.1016/j.postharvbio.2022.111915

[35] Cáceres D Díaz M Shinya P Infante R. Assessment of peach internal flesh browning through colorimetric measures. Postharvest Biol Technol. (2016) 111:48–52. doi: 10.1016/j.postharvbio.2015.07.007

[36] González-Buesa J Arias E Salvador M Oria R Ferrer-Mairal A. Suitability for minimal processing of non-melting clingstone peaches. Int J Food Sci Technol. (2011) 46:819–6. doi: 10.1111/j.1365-2621.2011.02572.x

[37] Arias E González J López-Buesa P Oria R. Optimization of processing of fresh-cut pear. J Sci Food Agric. (2008) 88:1755–63. doi: 10.1002/jsfa.3276

[38] Rocha A Morais A. Polyphenoloxidase activity and total phenolic content as related to browning of minimally processed ‘Jonagored’ apple. J Sci Food Agric. (2002) 82:120–6. doi: 10.1002/jsfa.1006

[39] Caglar H Paydas S. Changes of quality characteristics and aroma compounds of hybrids and some strawberry cultivars during harvest periods. Acta Hortic. (2002) 567:203–6. doi: 10.17660/actahortic.2002.567.40

[40] Zhu D Ren XJ Wei LW Cao XH Ge Y Liu H. Collaborative analysis on difference of apple fruits flavour using electronic nose and electronic tongue. Sci Hortic. (2020) 260:108879. doi: 10.1016/j.scienta.2019.108879

[41] Sevillano L Sanchez-Ballesta MT Romojaro F Flores FB. Physiological, hormonal and molecular mechanisms regulating chilling injury in horticultural species. Postharvest technologies applied to reduce its impact. J Sci Food Agric. (2009) 89:555–3. doi: 10.1002/jsfa.3468

[42] Jin P Zhu H Wang L Shan TM Zheng YH. Oxalic acid alleviates chilling injury in peach fruit by regulating energy metabolism and fatty acid contents. Food Chem. (2014) 161:87–93. doi: 10.1016/j.foodchem.2014.03.103, PMID: 24837925

[43] Crisosto CH Mitchell FG Ju Z. Susceptibility to chilling injury of peach, nectarine, and plum cultivars grown in California. HortScience. (1999) 34:1116–8. doi: 10.21273/HORTSCI.34.6.1116

[44] Crisosto CH Johnson RS DeJong T Day KR. Orchard factors affecting postharvest stone fruit quality. HortScience. (1997) 32:820–3. doi: 10.21273/HORTSCI.32.5.820

[45] Xi WP Zhang B Shen J Sun CD Xu CJ Chen KS. Intermittent warming alleviated the loss of peach fruit aroma-related esters by regulation of AAT during cold storage. Postharvest Biol Technol. (2012) 74:42–8. doi: 10.1016/j.postharvbio.2012.07.003

[46] El Hadi M Zhang F Wu F Zhou C Tao J. Advances in fruit aroma volatile research. Molecules. (2013) 18:8200–29. doi: 10.3390/molecules18078200, PMID: 23852166 PMC6270112

[47] Yang C Duan W Xie K Ren C Zhu C Chen K. Effect of salicylic acid treatment on sensory quality, flavor-related chemicals and gene expression in peach fruit after cold storage. Postharvest Biol Technol. (2020) 161:111089. doi: 10.1016/j.postharvbio.2019.111089

[48] Kakiuchi N Ohmiya A. Changes in the composition and content of volatile constituents in peach fruits in relation to maturity at harvest and artificial ripening. J Jpn Socifor Hortic Sci. (1991) 60:209–6. doi: 10.2503/jjshs.60.209

[49] Zhang B Shen J Wei W Xi W Xu C Ferguson I. Expression of genes associated with aroma formation derived from the fatty acid pathway during peach fruit ripening. J Agric Food Chem. (2010) 58:6157–65. doi: 10.1021/jf100172e, PMID: 20415420

[50] Fan X Zhao H Wang X Cao J Jiang W. Sugar and organic acid composition of apricot and their contribution to sensory quality and consumer satisfaction. Sci Hortic. (2017) 225:553–12. doi: 10.1016/j.scienta.2017.07.016

[51] Cao X Xie K Duan W Zhu Y Liu M Chen K. Peach carboxylesterase PpCXE1 is associated with catabolism of volatile esters. J Agric Food Chem. (2019) 67:5189–96. doi: 10.1021/acs.jafc.9b01166, PMID: 30997798

[52] Zhang B Xi WP Wei WW Shen JY Ferguson I Chen KS. Changes in aroma-related volatiles and gene expression during low temperature storage and subsequent shelf-life of peach fruit. Postharvest Biol Technol. (2011) 60:7–16. doi: 10.1016/j.postharvbio.2010.09.012

[53] Zhu X Luo J Li Q Li J Liu T Wang R. Low temperature storage reduces aroma-related volatiles production during shelf-life of banana fruit mainly by regulating key genes involved in volatile biosynthetic pathways. Postharvest Biol Technol. (2018) 146:68–78. doi: 10.1016/j.postharvbio.2018.08.015

[54] Altisent R Echeverria G Graell J Lopez L Lara I. Lipoxygenase activity is involved in the regeneration of volatile ester-synthesizing capacity after ultra-low oxygen storage of’ Fuji’ apple. J Agric Food Chem. (2009) 57:4305–12. doi: 10.1021/jf803930j 19378945

[55] Lara I Miró RM Fuentes T Sayez G Graell J López ML. Biosynthesis of volatile aroma compounds in pear fruit stored under long-term controlled-atmosphere conditions. Postharvest Biol Technol. (2003) 29:29–39. doi: 10.1016/S0925-5214(02)00230-2

[56] Avila CA Arévalo-Soliz LM Lingling J Navarre D Zhaorigetu C. Loss of function of FATTY ACID DESATURASE7 in tomato enhances basal aphid resistance in a salicylate-dependent manner. Plant Physiol. (2012) 158:2028–41. doi: 10.1104/pp.111.191262, PMID: 22291202 PMC3320204

[57] Ben Jemaa M Falleh H Serairi R Neves MA Snoussi M Isoda H. Nanoencapsulated *Thymus capitatus* essential oil as natural preservative. Innov Food Sci Emerg Technol. (2018) 45:92–7. doi: 10.1016/j.ifset.2017.08.017

[58] Karoui R Hassoun A. Efficiency of rosemary and basil essential oils on the shelf-life extension of Atlantic mackerel (*Scomber Scombrus*) fillets stored at 2°C. J AOAC Int. (2017) 100:335–4. doi: 10.5740/jaoacint.16-0410, PMID: 28074741

[59] Tabassum N Khan MA. Modified atmosphere packaging of fresh-cut papaya using alginate based edible coating: quality evaluation and shelf life study. Sci Hortic. (2020) 259:108853. doi: 10.1016/j.scienta.2019.108853

