# Supplementary figures and images for: Cinnamon essential oil vapor alleviates the reduction of aroma-related volatiles in cold-stored “Feicheng” peach using HS-GC-IMS
Source: Front Nutr. 2023 Jul 5;10:1122534. doi: 10.3389/fnut.2023.1122534 (PMC10354291; doi:10.3389/fnut.2023.1122534)

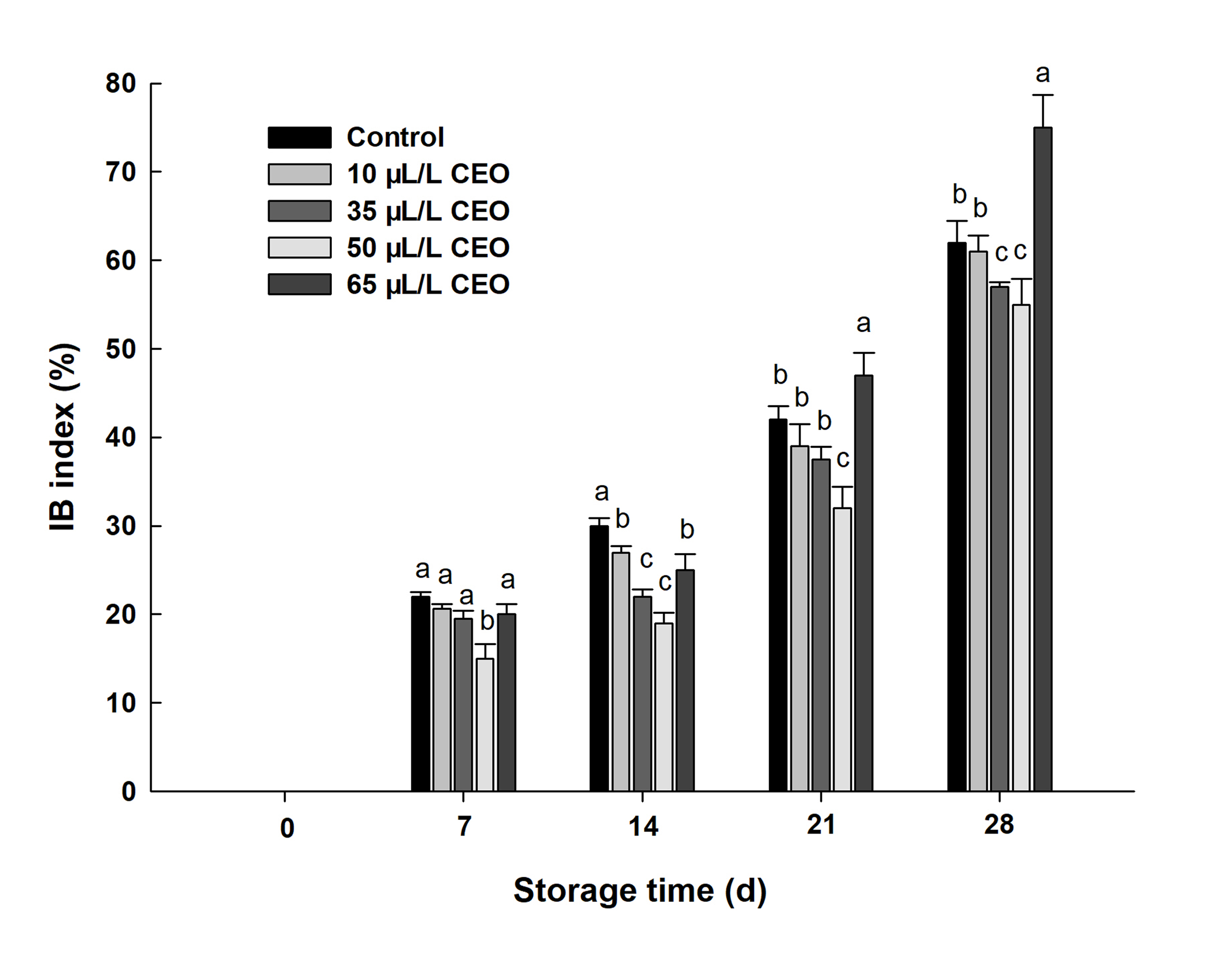

Supplement: Supplementary file 1 [file Image_1.JPEG]
